# Virus infection decreases the attractiveness of white clover plants for a non-vectoring herbivore

**DOI:** 10.1007/s00442-012-2322-z

**Published:** 2012-04-17

**Authors:** Tamara van Molken, Hannie de Caluwe, Cornelis A. Hordijk, Antonio Leon-Reyes, Tjeerd A. L. Snoeren, Nicole M. van Dam, Josef F. Stuefer

**Affiliations:** 1 Department of Experimental Plant Ecology, Radboud University, Heyendaalseweg 135, 6525 AJ Nijmegen, The Netherlands; 2 Department of Agriculture and Ecology, University of Copenhagen, Rolighedsvej 21, 1958 Frederiksberg C, Denmark; 3 Netherlands Institute of Ecology, NIOO-KNAW, Droevendaalsesteeg 10, 6708 PB Wageningen, The Netherlands; 4 Department of Plant–Microbe Interactions, Utrecht University, Padualaan 8, 3584 CH Utrecht, The Netherlands; 5 Present Address: Colegio de Agricultura, Universidad San Francisco de Quito, P.O. Box 17-1200-841, Quito, Ecuador; 6 Laboratory of Entomology, Wageningen University, Droevendaalsesteeg 1, 6708 PB Wageningen, The Netherlands; 7 Present Address: Nickerson-Zwaan BV, P.O. Box28, 4920 AA Made, The Netherlands; 8 Ecogenomics, Radboud University, Heyendaalseweg 135, 6525 AJ Nijmegen, The Netherlands; 9 Netherlands Organization for Scientific Research, P.O. Box 93510, 2509 AM Den Haag, The Netherlands

**Keywords:** Pathogens, Three-way interactions, *Trifolium repens*, Volatiles, White clover mosaic virus

## Abstract

Plant pathogens and insect herbivores are prone to share hosts under natural conditions. Consequently, pathogen-induced changes in the host plant can affect herbivory, and vice versa. Even though plant viruses are ubiquitous in the field, little is known about plant-mediated interactions between viruses and non-vectoring herbivores. We investigated the effects of virus infection on subsequent infestation by a non-vectoring herbivore in a natural genotype of *Trifolium repens* (white clover). We tested whether infection with *White clover mosaic virus* (WClMV) alters (1) the effects of fungus gnat feeding on plant growth, (2) the attractiveness of white clover for adult fungus gnat females, and (3) the volatile emission of white clover plants. We observed only marginal effects of WClMV infection on the interaction between fungus gnat larvae and white clover. However, adult fungus gnat females clearly preferred non-infected over WClMV-infected plants. Non-infected and virus-infected plants could easily be discriminated based on their volatile blends, suggesting that the preference of fungus gnats for non-infected plants may be mediated by virus-induced changes in volatile emissions. The compound β-caryophyllene was exclusively detected in the headspace of virus-infected plants and may hence be particularly important for the preference of fungus gnat females. Our results demonstrate that WClMV infection can decrease the attractiveness of white clover plants for fungus gnat females. This suggests that virus infections may contribute to protecting their hosts by decreasing herbivore infestation rates. Consequently, it is conceivable that viruses play a more beneficial role in plant–herbivore interactions than generally thought.

**Electronic supplementary material:**

The online version of this article (doi:10.1007/s00442-012-2322-z) contains supplementary material, which is available to authorized users.

## Introduction

Under natural conditions, plants are likely to be exposed to simultaneous attacks by pathogens and insect herbivores. Consequently, interactions between pathogens and herbivores sharing the same host plant are also likely to occur. Different studies have shown that the plant plays a central role in mediating such interactions, whereby pathogen-induced changes in host plants can affect herbivory, and vice versa (Hatcher and Paul 2000a; Rostás et al. 2003; Stout et al. 2006; Pieterse and Dicke 2007; De Vos and Jander 2010; Mouttet et al. 2011). Recent advances in genomics, plant physiology, and ecology have greatly enhanced our understanding of the interactions between plants, pathogens, and herbivores. It has become clear that pathogen infections in the host plant can both decrease (Gibbs 1980; Hatcher et al. 1994; Stout et al. 1999; De Vos et al. 2007; Röder et al. 2007; Rayapuram and Baldwin 2008; Thaler et al. 2010) and increase (Ericson and Wennström 1997; Moran 1998; Preston et al. 1999; Kruess 2002; Johnson et al. 2003; Belliure et al. 2005, 2010; Eubanks et al. 2005; Jiu et al. 2007; Thaler et al. 2010; Mouttet et al. 2011) herbivore consumption, growth rate, survival, and fecundity as well as attractiveness of the shared host plant for oviposition and feeding (Stout et al. 2006). The effects of phytopathogens on plant–herbivore interactions depend largely on the specific organisms involved (Felton and Korth 2000).

So far most ecological studies on plant pathogen–herbivore interactions have focussed on phytopathogenic fungi (Moran 1998; Hatcher et al. 1995; Hatcher and Paul 2000b; Kruess 2002; Johnson et al. 2003; Rostás et al. 2003; Röder et al. 2008; Kurtz et al. 2010; Mouttet et al. 2011), and relatively little is known about the effects of viruses on plant–herbivore interactions, especially in non-crop plant species. Phytopathogenic viruses can be closely associated with specific herbivores on which they rely for transmission between host plants (vectors). In these cases, virus-induced changes in the host plant usually result in attraction of the vectoring insect herbivores (Eigenbrode et al. 2002; Jiménez-Martínez et al. 2004; Werner et al. 2009; Mauck et al. 2010), with either positive (Belliure et al. 2005) or negative (Mauck et al. 2010) effects on herbivore performance. However, virus-infected plants are bound to experience damage by non-vectoring herbivores as well. Plant-mediated interactions between viruses and non-vectoring insects may fundamentally differ from interactions between viruses and their vectoring herbivores, owing to possible competitive relationships between pathogens and non-vectoring herbivores.

Few studies have investigated plant virus–herbivore interactions for non-vectoring herbivores. Lin et al. (2008) studied the effects of *Tomato mosaic virus* infection in tomato plants on herbivory by the corn earworm, *Helicoverpa armigera*, but found no clear effects of virus infection on feeding or oviposition preferences by the herbivore. In another study, Belliure et al. (2010) showed that *Tomato spotted wilt virus* enhances survival and oviposition of spider mites in pepper plants, while the developmental rate of spider mites remained unaffected by virus infection. Thaler et al. (2010) showed that *Tobacco mosaic virus* increases growth of *Spodoptera exigua* caterpillars when feeding on infected tomato plants. All these studies focussed on agricultural crops. Consequently, we know very little about the effects of virus infections on herbivory in non-crop species.

**Fig. 1:**
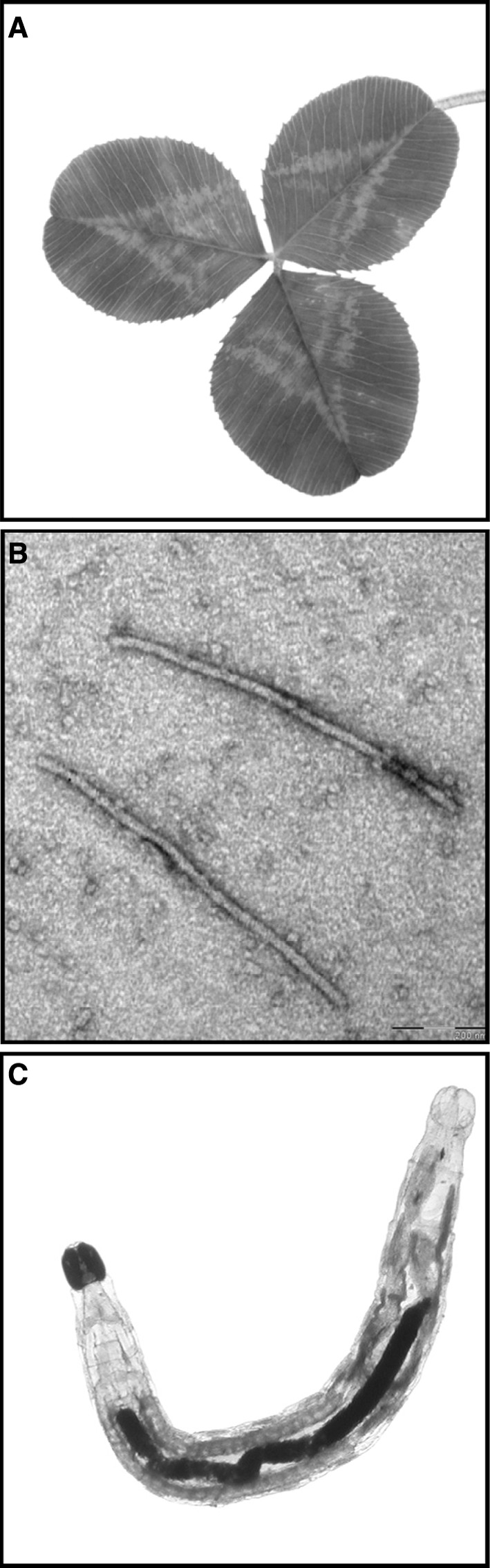
Model species used in this study: **a** a picture of a *Trifolium repens* leaf; **b** transmission electron microscopy image of *White clover mosaic virus*; **c** a fourth instar *Bradysia* sp. larva

In this paper, we investigated the effects of virus infection on subsequent attack by a non-vectoring insect herbivore in the stoloniferous herb *Trifolium repens* (white clover; Fig. 1a). *T. repens* is susceptible to *White clover mosaic virus* (WClMV; Fig. 1b) which was first reported in white clover by Pierce in 1935. WClMV occurs worldwide in temperate regions and can commonly be found in *T. repens* pastures (Sherwood 1997; Norton and Johnstone 1998; Coutts and Jones 2002; Denny and Guy 2009). The virus is not transmitted by insect vectors and spreads between host plants via mechanical transmission (Tapio 1970). WClMV can severely diminish biomass accumulation and decrease vegetative propagation in *T. repens* (Jones 1992; Dudas et al. 1998; Van Mölken and Stuefer 2011), although these effects may differ greatly among host genotypes (Van Mölken and Stuefer 2011). Previous greenhouse studies have provided circumstantial evidence for interactive effects of WClMV and larvae of non-vectoring fungus gnats (*Bradysia* sp.; Fig. 1c) on the growth of *T. repens* plants (Van Mölken, unpublished results). *Bradysia* sp. is widespread on all continents and inhabits a broad range of habitats (Menzel et al. 2003), including *T. repens* pastures (Springer and Carlton 1993).

To study the tripartite interaction between WClMV, fungus gnats, and their host plant *T. repens*, we first tested the hypothesis that WClMV infection alters the effects of fungus gnat infestation on *T. repens* growth. Second, we determined whether WClMV infection alters the attractiveness of *T. repens* to adult fungus gnats. This may be caused by virus-induced alterations in the composition of the volatile blend emitted by host plants (Eigenbrode et al. 2002; Jiménez-Martínez et al. 2004; Mauck et al. 2010). Therefore, we determined whether WClMV induces changes in the volatile composition of *T. repens* plants. Herbivorous insects can effectively discriminate between different bouquets of volatile organic compounds (VOCs; Smart and Blight 1997; Mewis et al. 2002; Quiroz et al. 2005; van Dam 2009), and chemical cues are predominantly responsible for the selection of oviposition sites by female herbivores (Renwick 1989).

The results of these experiments provide information about the possible influence of virus infections on the interaction between a non-vectoring insect herbivore and a non-crop plant species suggesting that, under certain conditions, host plants may indirectly benefit from virus infections (Roossinck 2011).

## Materials and methods

### Study organisms and experimental conditions

*Trifolium repens* L. (white clover) and *White clover mosaic virus* (WClMV) were used for all experiments. *T. repens* produces clonal offspring (ramets) which develop at the nodes of horizontally growing stems (stolons). Each individual ramet consists of a single leaf, an internode, and meristems which can develop into roots, branches, and flowers. *T. repens* is an important plant for pastures and hence cultivated worldwide. However, we performed all experiments with cuttings from a single genotype (A120), which had been collected from a wild population (riverine grasslands) and grown under common garden and greenhouse conditions for 5 years before we conducted this experiment (see Weijschede et al. 2006 for details). This genotype was previously shown to respond most strongly to WClMV infection in terms of reduced growth, as compared to ten other genotypes (Van Mölken and Stuefer 2011). Total plant biomass and the number of ramets were used as an indication of plant fitness, since they are considered the best fitness proxies in plants with reduced (or no) sexual reproduction (Sackville-Hamilton et al. 1987; Winkler and Fischer 1999; Pan and Price 2002). All experiments (unless stated differently) were carried out in a greenhouse with a 16/8 h light/dark period, at 19/18 °C.

WClMV (necrosis strain, originally isolated from *T. repens*) was obtained from the Deutsche Sammlung von Mikroorganismen und Zellkulturen (Braunschweig, Germany). Plants in the virus treatments were mechanically inoculated with infected cell sap, prepared by grinding WClMV-infected plant material in inoculation buffer (50 mM Na_2_HPO_4_ buffer, 1 mM EDTA, set to pH 7.0 with HCl). Leaves were dusted with carborundum (500 mesh), and 10 μl virus suspension was rubbed on each leaf by hand. Control plants were mock-inoculated with inoculation buffer only. WClMV presence was tested by DAS-ELISA (Clark and Adams 1977). Virus replication starts within a few hours after inoculation (Bos 1999) after which a systemic infection will be established (Van Mölken et al. 2011). The infection process is characterized by a rapid multiplication of virus particles within the whole plant. In *Phaseolus vulgaris*, for instance, the concentration of WClMV shows a more than 10-fold increase within 7 days post-inoculation (Clarke, Burritt and Guy 1998). The rate of multiplication depends on various factors such as the rate and stage of plant development, temperature, and light availability, implying that after a few weeks the concentration of virus particles in the plant is virtually independent of the virus concentration in the inoculum.

Adult fungus gnats (*Bradysia* sp.; Sciaridae) were collected from the greenhouse and maintained in the laboratory under controlled conditions. Adult female fungus gnats oviposit into the soil and the emerging larvae feed on organic soil matter, and young roots. Fungus gnats are pervasive greenhouse pests (Harris et al. 1996) and common in nature (Menzel et al. 2006; Kjaerandsen et al. 2007; Vilkamaa, Salmela and Hippa 2007). The collected adults were placed in 1-l plastic pots containing moist potting soil mixed with rabbit food (2:1/v:v; Hope farms, Woerden, the Netherlands) and allowed to propagate. Naive fungus gnats that had never been exposed to plant tissue were used for all experiments.

### Effects of virus infection and fungus gnat feeding on plant performance

To study combined effects of fungus gnats and virus infection on plant growth, 64 cuttings were individually planted in plastic trays (16 × 12 × 5 cm) filled with sterilized clay grains (SERAMIS; Masterfoods, Germany) and placed in a climate chamber with a 16/8 h light/dark period at 20/18 °C. Each cutting consisted of an apical meristem and four rooted nodes. Each of the nodes had a single leaf attached to it. Plants of the same fungus gnat treatment were put into mesh cages to avoid uncontrolled contamination with fungus gnats. The first two fully expanded leaves were used for mechanical inoculation. Fungus gnats and WClMV were applied in a full-factorial design, resulting in 16 replicate plants per treatment. Cuttings assigned to the infection treatment were mechanically inoculated 7 days after planting. We allowed the virus infection to establish for another 5 days before adding the first fungus gnat larva. The larval period usually lasts up to 14 days (Berg 2000), which is considerably shorter than the time span of the experiment. To ensure continuous herbivore pressure throughout the experiment, we applied larvae at four different points in time. A total of 50 larvae (3rd and 4th instars) were applied. Twenty fungus gnat larvae were applied to the substrate of each individual plant (no-choice) in the fungus gnat treatment on 6 and 13 days post-inoculation. Another five larvae per plant were added at 20 and 31 days post-inoculation. Due to the short time span of the larval period, an average of 21 larvae were present in each individual plant tray during the experiment. This herbivore pressure falls within the range of larvae observed in the field. According to Frouz (1999), the number of Sciaridae larvae in arable land, fallows, and grasslands ranges from 13 to 1,358 individuals per square meter, and the author suggests that these numbers may underestimate actual field values. These numbers would correspond roughly to 0.25–26 individuals per tray in our experiment. *Bradysia* females lay on average 120 eggs during their lifespan (Berg 2000, and references therein) indicating that larval pressure may locally become very high in *Bradysia*-prone environments. At 36 days post-inoculation, the length of the primary stolon was measured, and the total number of ramets and the number of branches were counted. All plant material was dried at 70 °C for 72 h, and dry weights of roots, stolons, and leaves were measured separately.

### Effects of virus infection on plant attractiveness to fungus gnats

To determine whether adult fungus gnats prefer healthy over virus-infected plants, we carried out choice tests with adult females of *Bradysia*. We planted uniform individual cuttings in plastic pots (7 × 7 × 8 cm). Cuttings consisted of an apical meristem and four rooted nodes holding a single leaf each. For practical reasons, cuttings in the virus treatment were inoculated with WClMV on the first two fully expanded leaves at 18 and 3 days prior to the analysis for the first and second set of replicates, respectively. The results from both sets were very similar (*χ* ^2^ test, effect of day on fungus gnat preference, *χ* ^2^ = 0.00, *p* = 0.9747) and were pooled in the final analysis. To allow for sufficient volatile emission, we placed three pots with plants of the same treatment together. For each treatment, the cuttings were placed in a 5-l glass cuvette at the upwind end of one of the two arms of a closed-system Y-tube olfactometer (Takabayashi and Dicke 1992), and established a constant air flow towards the lower part of the tube. Then, 4 l min^−1^ of air (filtered through activated charcoal) was driven through each olfactometer arm and pulled towards the base of the olfactometer by the laboratory vacuum system at 8 l min^−1^. After an acclimation period of 30 min, an individual female fungus gnat was placed at the downwind end of the olfactometer and observed for a maximum of 10 min. The choice was recorded when the insect remained in the upwind end of one of the arms for more than 20 s, or if it remained in the middle part (5 cm upwind of the junction) of one of the arms for more than 50 s. These criteria are analogous to those used in similar studies (Pareja et al. 2007; Yin-Quan et al. 2009; Najar-Rodriguez et al. 2010; Addesso et al. 2011).

For each replicate set of virus-infected and non-infected plants, we recorded the preference of 8–10 female fungus gnats as described above. After that, we replaced the plants and started the next choice test with a new batch of females. In total, we tested seven sets of plants in this manner. We consider each set of virus-infected and non-infected plants as a replicate, since fungus gnats preference within one set of plants cannot be considered statistically independent. Each fungus gnat and plant was used only once. Six fungus gnats did not make a choice within 10 min and were excluded from subsequent analyses. The experiments were carried out at 24 °C.

### Virus induced changes in plant volatile emission

To analyze volatile production of virus-infected and non-infected plants, we planted individual cuttings of four ramets in plastic pots (7 × 7 × 8 cm). Cuttings in the virus treatment were inoculated with WClMV on the first two fully expanded leaves 4 days prior to the analysis. Each replicate consisted of four independent cuttings grouped together during the measurements. Each cutting was used once. The sampling was repeated 13 times per treatment. VOC collection, desorption, and identification were performed according to the protocol of Soler et al. (2007) with the following modifications: the glass collection chambers containing the plants were constantly supplied at the top with 150 ml min^−1^ of pressurized air (Hoekloos, NL) cleaned over a Zero Air generator to remove hydrocarbons (Parker Hannifin, Tewksbury, MA, USA). The headspace was collected for 80 min in steel traps filled with 150 mg Tenax TA and 150 mg Carbopack B, and analyzed after direct desorption with a GC–MS system. We measured five replicates per treatment and two background VOC profiles from pots filled with substrate in parallel sessions. The background sample peak area was subtracted from the plant sample peak area for final analyses. The whole procedure was repeated with an additional eight replicates per treatment and one background VOC profile. Volatiles were desorbed from the traps using an automated thermodesorption unit (model Unity; Markes, Llantrisant, UK) at 200 °C for 12 min and focused on a cold Tenax trap (−10 °C). After 1 min of dry purging, trapped volatiles were introduced into the GC–MS (model Trace; ThermoFinnigan, Austin, TX, USA) by heating the cold trap for 3 min to 270 °C (split ratio 1:4; column 30 m × 0.32 mm ID RTX-5 Silms; film thickness 0.33 μm). The volatiles were detected by the MS operating at 70 eV in EI mode. Mass spectra were acquired in full scan mode (33–300 AMU, 0.4 scan s^−1^). Compounds were identified by their mass spectra (Soler et al. 2007) using deconvolution software (AMDIS), in combination with Nist 98 and Wiley 7th edition spectral libraries, and by comparing their linear retention indices (RI). Additionally, mass spectra and/or linear retention indices of chromatographic peaks were compared with values reported in the literature. Further confirmation of compound identification was obtained by interpolating retention indices of homologous series and by comparing analytical data with those of reference substances (farnesene, ±limonene, methyljasmonate, methylsalicylate, dimethyldisulfide, dimethyltrisulfide, octanal, nonanal, decanal, cis-3-hexen-1-ol, 2-phenylethylalcohol, indole, and benzylcyanide; Sigma-Aldrich, St. Louis, MO, USA). The integrated signals generated by the AMDIS software from the MS-chromatograms were used for comparing treatments.

### Statistical analysis

Main and interaction effects of WClMV and fungus gnats on plant growth were analyzed by two-way ANOVA, the percentage of biomass allocation was log-transformed to meet the ANOVA assumptions. Similarities in the volatile blends between virus-infected and non-infected plants were analyzed by canonical discriminant analysis (CDA; Soler et al. 2007). To satisfy assumptions of CDA, a subset of 24 volatile compounds was selected for analysis for their role in plant–insect interactions. The selection was made based on the ubiquity in the samples. CDA allowed for the identification of compounds that discriminate most clearly between treatments. This analysis provides a ranking of compounds according to their power for discriminating VOC blends emitted from virus-infected and non-infected plants, respectively. High-ranking compounds correlate most closely with the first canonical axis and are hence most important for differentiating between the two groups. Simple correlation coefficients (total canonical structure) were used to quantify the association between compounds and the canonical axis. The probabilities of misclassification were estimated with the CROSS-VALIDATE option in SAS procedure DISCRIM. This approach reclassifies every data point as if it were a new observation, thereby reconstructing the discriminant function without that observation. Cohen’s κ (kappa) coefficient was calculated to assess the significance of classification agreement. A two-way ANOVA was carried out to test for the effect of virus infection on peak areas of each of the 24 selected compounds. Virus infection was used as main factor and sampling time as block effect. Assumptions of ANOVA were verified for each variable prior to analysis. The effect of virus infection on fungus gnat preference was analyzed by GEE analysis in proc GENMOD of SAS. Each replicate set of plants contained (1) the number of choices made for control versus virus-infected plant (events) and (2) the total number of trials. We analyzed the data with the events/trials response variable, thereby allowing for summarized binomial response data. The REPEATED statement was used to identify each replicate set of plants as a statistically independent subject, because responses within subjects were assumed to be correlated. Virus treatment was regarded as a within-subject factor. All tests were carried out with SAS, v.9.1 (SAS Institute, Cary, USA).

## Results

### Effects of virus infection and fungus gnat feeding on plant performance

Fungus gnat infestation caused a greater depression in the growth and development of host plants than did WClMV. WClMV infection (Table 1, virus effects) reduced total plant biomass production by 27 %. This reduction was almost twice as high in fungus gnat infested plants (52 %; Table 1, fungus gnats effects). Fungus gnats reduced the total number of ramets (44 %) and the percentage of branches on the main stolon (41 %), whereas these plant traits remained unaffected by virus infection.

**Table 1:** Statistical analysis of the effects of WClMV, fungus gnat larvae and their interaction (ANOVA) on different developmental and growth traits

Trait	Error		Virus				Fungus gnats				Virus × fungus gnats			
	*df*	MS	*df*	MS	*F*	*P*	*df*	MS	*F*	*P*	*df*	MS	*F*	*P*
Total no. of ramets	59	70.16	1	191.18	2.72	0.1041	1	8,773.01	125.04	<0.0001	1	2.81	0.04	0.8419
Length of pr. stolon	59	16.10	1	242.76	15.08	0.0003	1	212.11	13.18	0.0006	1	26.49	1.65	0.2045
% Branches on pr. stolon	59	136.44	1	243.73	1.79	0.1865	1	8,949.87	65.59	<0.0001	1	153.28	1.12	0.2935
Total biomass	59	0.05	1	1.02	22.00	<0.0001	1	5.74	124.36	<0.0001	1	0.24	5.13	0.0272
Biomass of roots	59	0.00	1	0.02	12.18	0.0009	1	0.24	121.67	<0.0001	1	0.00	0.33	0.5658
Biomass of stolons	59	0.01	1	0.17	27.41	<0.0001	1	0.47	76.6	<0.0001	1	0.04	5.94	0.0178
Biomass of leaves	59	0.01	1	0.20	18.05	<0.0001	1	1.5	136.88	<0.0001	1	0.07	6.7	0.0121
Biomass per ramet	59	0.00	1	0.00	14.89	0.0003	1	0.00	10.53	0.0019	1	0.00	5.15	0.0269
Root–shoot ratio	59	0.00	1	0.00	0.32	0.5736	1	0.00	0.34	0.5624	1	0.02	11.63	0.0012
% Biomass of roots	59	6.88	1	2.16	0.31	0.5771	1	2.90	0.42	0.5190	1	80.62	11.72	0.0011
% Biomass of stolons	59	0.00	1	0.00	3.43	0.0692	1	0.05	35.74	<0.0001	1	0.00	2.97	0.0898
% Biomass of leaves	59	10.57	1	13.19	1.25	0.2686	1	256.11	24.22	<0.0001	1	19.88	1.88	0.1755

*Pr. stolon* the primary stolon, *df* degrees of freedom, *MS* mean square, *F* F statistic

**Fig. 2:**
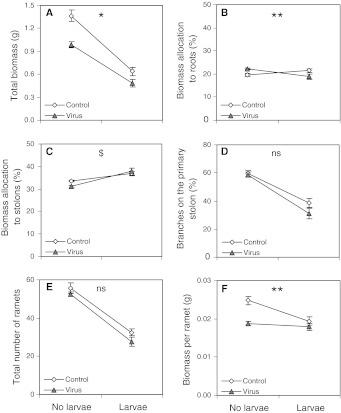
Interaction figures showing the effects of virus infection and fungus gnat herbivory on **a** total plant biomass, **b** percentage of biomass allocation to the roots, **c** percentage of biomass allocation to the stolons, **d** percentage of branches on the primary stolon, **e** total number of ramets, **f** biomass per ramet. *Error bars* standard errors. For the interaction term (2-way ANOVA): *ns* not significant, ^$^ *p* > 0.05, <0.1, *p < 0.05, **p < 0.01

For a number of traits, the effects of fungus gnats on plant growth differed slightly between non-infected and WClMV-infected plants (Table 1, virus × fungus gnat interaction). For example, the reduction in total biomass production caused by fungus gnats was slightly less pronounced in virus-infected plants than in non-infected plants (51 vs. 53 %, respectively; Fig. 2a). Fungus gnat infestation increased proportional biomass allocation to roots in non-infected plants, but decreased this allocation in virus-infected plants (Fig. 2b). There was no significant interaction between virus infection and fungus gnat infestation with respect to proportional biomass allocation to stolons (Fig. 2c) or leaves, the branching percentage on the primary stolon (Fig. 2d), or the total number of ramets (Fig. 2e). However, plant biomass investment per ramet (Fig. 2f) was strongly reduced by fungus gnats in non-infected plants (22 %), but showed only a slight decrease in virus-infected plants (5 %). Mean values ± SE of various developmental and growth traits in each of the treatments are given in supplementary Table 1.

### Effects of virus infection on plant attractiveness to fungus gnats

**Fig. 3:**
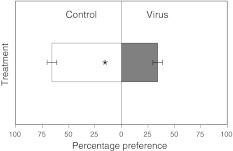
Fungus gnats show a preference for non-infected (*Control*) as compared to *Virus*-infected plants. We counted the number of fungus gnats that preferred non-infected and virus-infected plants and expressed this as a percentage of the total number of trials per plant pair (for statistical procedure see “Materials and Methods”). *Bars* give mean percentage of adult female fungus gnats which preferred non-infected or virus-infected plants, respectively, as determined in dual choice tests. *Error bars* standard errors, *p < 0.05

Virus infection had a strong effect on the host plant’s attractiveness for adult female fungus gnats. These adults clearly preferred non-infected plants (67 %) over virus-infected plants (33 %), the preference being expressed as a percentage choice for either one of the two plant groups. This shows that fungus gnat adults can discriminate between the two plant groups based on the different emissions of VOCs (*χ* ^2^ = 4.84, *p* = 0.0278; Fig. 3 in favor of the healthy plants.

### Virus-induced changes in plant volatile emission

**Fig. 4:**
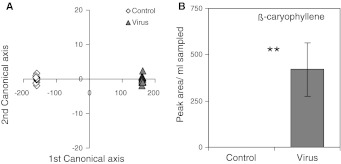
Difference in volatile blends between virus-infected and non-infected plants. **a** Results of a canonical discrimination analysis between volatile blends emitted by non-infected and virus-infected plants, respectively. Non-infected and virus-infected plants can be discriminated on the basis of their volatile blends, indicating that virus infection alters the emission of volatile compounds. The analysis is based on 24 selected volatile compounds. **b** Emission of β-caryophyllene increases with virus infection, and this compound is not emitted by non-infected plants. *Error bars* standard errors, **p < 0.01

Control and infected plants emitted significantly different volatile blends (κ = 0.9231; *p* = < 0.0001; Fig. 4a). A more detailed analysis of the compounds (Table 2) shows that only two individual compounds differed significantly between virus-infected and non-infected plants. The emission of both β-caryophyllene (*F* = 11.39, *p* = 0.0027), which was most important for the discrimination between the two groups (rank 1 for total canonical structure; Table 2), and benzonitrile (*F* = 4.59, *p* = 0.0435) increased with virus infection of the plant. Strikingly, β-caryophyllene was exclusively detected in the headspace of virus-infected plants and was not emitted by control plants (Fig. 4b). Thus, virus-infected plants emitted unique volatile blends that differed from those emitted by uninfected plants.

**Table 2:** Volatile compounds emitted by virus-infected and non-infected plants

Compound	RI	TCS	Rank	Mean peak area ml^−1^ sampled ± SE		ANOVA	
				Control	Virus	*F*	*P*
**β-Caryophyllene**	**1,412**	**0.513**	**1**	**0** **±** **0**	**420** **±** **144**	**11.39**	**0.0027**
1-Hexadecanol	1,882	0.201	2	4,872 ± 2,421	97,496 ± 92,185	1.66	0.2113
2-Nonanone	1,091	0.174	3	116 ± 51	196 ± 77	0.30	0.5907
**Benzonitrile**	**978**	**0.174**	**4**	**682** **±** **203**	**963** **±** **252**	**4.59**	**0.0435**
3-Hexen-1-ol-acetate (mix of *E* and *Z*)	1,008	0.139		8,331 ± 3,114	13,154 ± 6,282	1.13	0.2983
Pentanoic acid	880	0.116	6	717 ± 224	946 ± 334	0.21	0.6501
Dimethyldisulfide	738	0.098	7	449 ± 88	515 ± 105	0.06	0.8115
Acetic acid	569	0.094	8	4,632 ± 1,839	5,985 ± 2,255	0.48	0.4948
Ethylbenzene	857	0.072	9	17,019 ± 6,521	20,452 ± 7,242	0.02	0.8811
Methyl-cyclohexane	720	0.046	10	1,324 ± 454	1,473 ± 470	0.00	0.9995
2-Ethyl-1-hexanol	1,032	0.041	11	24,998 ± 8,140	27,530 ± 9,620	0.00	0.9777
Menthol isomer	1,174	0.034	12	1,916 ± 857	2,091 ± 592	0.14	0.7137
Acetic acid butyl ester	804	0.030	13	1,127 ± 487	1,234 ± 556	0.00	0.9980
Propyl-cyclohexane	921	−0.002	14	132 ± 37	132 ± 48	0.04	0.8413
α-Pinene	927	−0.023	15	1,344 ± 406	1,282 ± 353	0.24	0.6276
2-Propanol-1-butoxy	938	−0.056	16	4,234 ± 1,438	3,678 ± 1,440	0.21	0.6481
Limonene	1,027	−0.133	17	6,171 ± 1,742	4,424 ± 2,004	0.75	0.3961
1-Butanol	658	−0.169	18	5,022 ± 2,166	2,945 ± 1,197	1.30	0.2663
2-Hexenal (*E*)	841	−0.182	19	1,446 ± 552	880 ± 292	0.40	0.5339
Phenylethyl alcohol	1,106	−0.196	20	357 ± 113	218 ± 86	1.33	0.2606
Ethyl-cyclohexane	823	−0.211	21	787 ± 312	426 ± 135	2.22	0.1508
2-Methyl-1.3-butadiene	522	−0.218	22	3,058 ± 1,119	1,684 ± 573	3.03	0.0956
α-Copaene	1,371	−0.228	23	420 ± 157	209 ± 96	2.74	0.1120
2-β-pinene	970	−0.229	24	626 ± 240	316 ± 121	3.77	0.0650

The linear retention indices (*RI*) used for identification of the compounds are given in the first column. The total canonical structure (*TCS*) provides correlation coefficients between each compound and the first canonical axis, indicating the relative power of each compound for discriminating between volatile blends emitted by virus-infected and non-infected plants. The compounds are ranked according to their TCS values. Mean peak areas ml^−1^ sampled air are given for each compound in the control and the virus treatment, respectively. The last two columns give results of univariate ANOVA tests, comparing the concentrations of each compound in the control and virus treatment. Significant differences are indicated in bold. β-caryophyllene is the most important compound for discriminating between virus-infected and non-infected plants (highest TCS and rank) and the emission of this compound was altered significantly by virus-infection

## Discussion

Plant-mediated interactions between pathogens and herbivores may occur when pathogen-induced changes in the host affect herbivory. We demonstrate that, in *Trifolium repens*, WClMV decreases the attractiveness of the host plant for adult fungus gnat females. This suggests that WClMV infection may reduce fungus gnat infestation rates when infected host plants co-occur with non-infected plants.

Within the context of this experiment, fungus gnat feeding strongly affected total plant biomass and this effect was two times greater than biomass losses caused by WClMV infection alone. Furthermore, while WClMV showed no effect on the total number of ramets, fungus gnat infestation drastically decreased the number of ramets produced by their hosts. The total number of ramets and the total biomass are important traits because they are one of the best proxies for performance in clonal plants that show limited sexual reproduction. Thus, fungus gnats can have clear negative effects on plant performance. However, the reduction in total number of ramets caused by fungus gnats did not differ between non-infected and virus-infected plants. Furthermore, the reduction in biomass production caused by fungus gnats was only marginally less in virus-infected plants than in non-infected plants. In other words, effects of fungus gnats on *T. repens* growth were not greatly affected by WClMV infection. In our experiment, the larvae were confined to individual host plants, preventing possible effects of larval preference for virus-infected versus non-infected plants. Schmelz et al. (2002) have shown that fungus gnat larvae preferably feed on control *Spinacia oleracea* plants instead of herbivore-induced plants. In analogy, larval preference might result in differential consumption of virus-infected and non-infected plants in our system. A choice test between virus-infected and non-infected plants would be needed to directly confirm whether fungus gnat larvae can differentiate between these plants, and whether such differentiation correlates with the preference of adult female fungus gnats. The ecological importance of such selective feeding for plant growth remains questionable given the relatively low mobility of larvae as compared to fungus gnat adults.

The attractiveness of the host plant for female fungus gnats was significantly reduced by WClMV infection. Such virus-induced changes in insect preference have also been shown in other studies (Eigenbrode et al. 2002; Jiménez-Martínez et al. 2004; Werner et al. 2009; Mauck et al. 2010); however, our results differ from these studies in two important ways. First, those studies consider insects that serve as vectors for virus transmission between host plants, while we show that WClMV infection affected insects that have no clear ecological association with the virus. Second, in the above-mentioned studies, virus-infected plants attract the insect vectors which seems a logical consequence of the relationship between the plant virus and its vector. On the contrary, we show that adult fungus gnats prefer non-infected plants over virus-infected plants. Reduced oviposition in the soil surrounding virus-infected plants will likely result in benefits for the plant given the severe effects of fungus gnat larvae on plant growth and development, as observed in this study.

The WClMV-induced change in plant attractiveness for female fungus gnats is probably mediated by changes in volatile emission by the host plant. Our results suggest that this effect depends at least partly on the emission of β-caryophyllene. This compound was emitted only by virus-infected plants and was not observed in the volatile blend of non-infected plants. This finding is consistent with previous work (Werner et al. 2009) showing increased caryophyllene emission by *Potato leafroll virus*—infected potato plants at 2–8 weeks post-inoculation. β-Caryophyllene may thus play a role in plant attractiveness. However, further experiments testing the repelling function of isolated compounds are needed to confirm this hypothesis. Jasmonic acid (JA) could also play a role in this process as JA activity increases in response to WClMV infection in plants such as *Phaseolus vulgaris*, reaching a plateau within 5 days post-inoculation (Clarke et al. 2000). It is unknown whether JA activity increases in *T. repens* plants upon WClMV infection. Nevertheless, JA induction may play a role in white clover resistance to herbivores, considering that increased JA levels are known to stimulate the biosynthesis of β-caryophyllene (Thaler et al. 2002; Arimura et al. 2008).

There may be several reasons why herbivores avoid infected plants. First, herbivores may avoid damaged plants altogether, independent of which biotic or abiotic agents caused the damage. Alternatively, pathogen infection can decrease the nutritional value of the plant tissue (Wroth et al. 1993; Hatcher et al. 1997; Mauck et al. 2010) and thus herbivores may benefit from avoiding infected host plants, since plants with a lower food quality would decrease their performance, fecundity, and offspring vigor (Awmack and Leather 2002; Mauck et al. 2010). Third, it is well known that herbivore-induced changes in plant volatile emissions can attract parasitoids both above- (Vet and Dicke 1992; Dicke 1999) and below-ground (Aratchige et al. 2004; Rasmann et al. 2005). β-Caryophyllene can serve as an indirect defence signal in herbivore-induced maize plants by attracting entomopathogenic nematodes (Rasmann et al. 2005). Since the same entomopathogenic nematodes also attack fungus gnat larvae (Saunders and Webster 2000), β-caryophyllene may serve as a cue for future parasitism.

### Ecological implications

The results in this paper suggest that there may be ecological benefits of virus infection for host plants in terms of virus-induced herbivore repellence. Such benefits may accrue if virus infections in plants result in protection of the host from subsequent herbivore attacks, and if the protection from herbivory outweighs performance losses due to virus infection. WClMV incidence can vary greatly within and between pastures of *T. repens* (Denny and Guy 2009), resulting in a mosaic of infected and non-infected plants which may elicit selective behavior by herbivores. Future studies measuring herbivore infestation rates on virus-infected and non-infected plants in the field can determine the consequences of herbivore preference for plant fitness. This would allow for a direct test of possible ecological benefits associated with virus infection in host plants.

Evidently, the generality of the results presented here and their relevance for natural conditions depends on the specific ecological context, i.e. the organism involved, the frequency and spatial scales at which pathogens and herbivores co-occur, the severity of their individual effects on plant performance, the order in which they attack the host plant, and the time span in which the interaction takes place. Nevertheless, our results support the notion that pathogen infections may play a role in plant–herbivore interactions. Our results provide a basis for testing pathogen-induced plant repellence against a range of herbivorous insects, and suggest that the consequences of virus-infections may ultimately depend on multiple interactions in the biotic environment of the host plant (Roossinck 2011).

## Electronic supplementary material

Below is the link to the electronic supplementary material.

Supplementary material 1 (DOC 62 kb)

## References

[CR1] Addesso KM, McAuslane HJ, Alborn HT (2011). Attraction of pepper weevil to volatiles from damaged pepper plants. Entomol Exp Appl.

[CR2] Aratchige NS, Lesna I, Sabelis MW (2004). Below-ground plant parts emit herbivore-induced volatiles: olfactory responses of a predatory mite to tulip bulbs infested by rust mites. Exp Appl Acarol.

[CR3] Arimura GI, Garms S, MaVei M, Bossi S, Schulze B, Leitner M, Mithöfer A, Boland W (2008). Herbivore-induced terpenoid emission in *Medicago truncatula*: concerted action of jasmonate, ethylene and calcium signaling. Planta.

[CR4] Awmack CS, Leather SR (2002). Host plant quality and fecundity in herbivorous insects. Annu Rev Entomol.

[CR5] Belliure B, Janssen A, Maris PC, Peters D, Sabelis MW (2005). Herbivore arthropods benefit from vectoring plant viruses. Ecol Lett.

[CR6] Belliure B, Sabelis MW, Janssen A (2010). Vector and virus induced plant responses that benefit a non-vector herbivore. Basic Appl Ecol.

[CR7] Berg MP (2000). Mass-length and mass-volume relationships of larvae of *Bradysia paupera* (Diptera: Sciaridae) in laboratory cultures. Eur J Soil Biol.

[CR8] Bos L (1999). Plant viruses, unique and intriguing pathogens. A textbook of plant virology.

[CR9] Clark MF, Adams AN (1977). Characteristics of the microplate method of enzyme-linked immunosorbent assay for detection of plant viruses. J Gen Virol.

[CR10] Clarke SF, Burritt DJ, Guy PL (1998). Influence of plant hormones in virus replication and pathogenesis-related proteins in *Phaseolus vulgaris* L infected with White clover mosaic potexvirus. Physiol Mol Plant Path.

[CR11] Clarke SF, Guy PL, Jameson PE, Schmierer D, Burritt DJ (2000). Influence of White clover mosaic potexvirus on the endogeneous levels of jasmonic acid and related compounds in *Phaseolus vulgaris* L seedlings. J Plant Physiol.

[CR12] Coutts BA, Jones RAC (2002). Temporal dynamics of spread of four viruses within mixed species perennial pastures. Ann Appl Biol.

[CR13] De Vos M, Jander G (2010). Volatile communication in plant–aphid interactions. Curr Opin Plant Biol.

[CR14] De Vos M, van Oosten VR, Jander G, Dicke M, Pieterse CMJ (2007). Plants under attack. Multiple interactions with insects and microbes. Plant Signal Behav.

[CR15] Denny BL, Guy PL (2009). Incidence and spread of viruses in white-clover pastures of the South Island, New Zealand. Australas Plant Pathol.

[CR16] Dicke M (1999). Are herbivore-induced plant volatiles reliable indicators of herbivore identity to foraging carnivorous arthropods?. Entomol Exp Appl.

[CR17] Dudas B, Woodfield DR, Tong PM, Nicholls MF, Cousins GR, Burgess R, White DWR, Beck DL, Lough TJ, Forster RLS (1998). Estimating the agronomic impact of white clover mosaic virus on white clover performance in the North Island of New Zealand. N Z J Agr Res.

[CR18] Eigenbrode SD, Ding H, Shiel P, Berger PH (2002). Volatiles from potato plants infected with Potato leafroll virus attract and arrest the virus vector, *Myzus persicae* (Homoptera: Aphididae). Proc R Soc Lond B.

[CR19] Ericson L, Wennström A (1997). The effect of herbivory on the interaction between the clonal plant *Trientalis europaea* and its smut fungus *Urocystis trientalis*. Oikos.

[CR20] Eubanks MD, Carr DE, Murphy JF (2005). Variation in the response of *Mimulus guttatus* (Scrophulariaceae) to herbivore and virus attack. Evol Ecol.

[CR21] Felton GW, Korth KL (2000). Trade-offs between pathogen and herbivore resistance. Curr Opin Plant Biol.

[CR22] Frouz J (1999). Use of soil dwelling Diptera (Insecta, Diptera) as bioindicators: a review of ecological requirements and response to disturbance. Agric Ecosyst Environ.

[CR23] Gibbs A (1980). A plant virus that partially protects its wild legume host against herbivores. Intervirology.

[CR24] Harris MA, Gardner WA, Oetting RD (1996). A review of the scientific literature on fungus gnats (Diptera: Sciaridae) in the genus *Bradysia*. J Entomol Sci.

[CR25] Hatcher PE, Paul ND (2000). Beetle grazing reduces natural infection of *Rumex obtusifolius* by fungal pathogens. New Phytol.

[CR26] Hatcher PE, Paul ND (2000). On integrating molecular and ecological studies of plant resistance: variety of mechanisms and breadth of antagonists. J Ecol.

[CR27] Hatcher PE, Paul ND, Ayres PG, Whittaker JB (1994). The effect of a foliar disease (rust) on the development of *Gastrophysa viridula* (Coleoptera: Chrysomelidae). Ecol Entomol.

[CR28] Hatcher PE, Paul ND, Ayres PG, Whittaker JB (1995). Interactions between *Rumex* spp, herbivores and a rust fungus: the effect of *Uromyces rumicis* infection on leaf nutritional quality. Funct Ecol.

[CR29] Hatcher PE, Paul ND, Ayres PG, Whittaker JB (1997). The effect of nitrogen fertilization and rust fungus infection, singly and combined, on the leaf chemical composition of *Rumex obtusifolius*. Funct Ecol.

[CR30] Jiménez-Martínez ES, Bosque-Pérez NA, Berger PH, Zemetra RS, Ding H, Eigenbrode SD (2004). Volatile cues influence the response of *Rhopalosiphum padi* (Homoptera: Aphididae) to Barley yellow dwarf virus–infected transgenic and untransformed wheat. Environ Entomol.

[CR31] Jiu M, Zhou X-P, Tong L, Xu J, Yang X, Wan F-H, Liu S-S (2007). Vector-virus mutualism accelerates population increase of an invasive whitefly. PLoS One.

[CR32] Johnson SN, Douglas AE, Woodward S, Hartley SE (2003). Microbial impacts on plant–herbivore interactions: the indirect effects of a birch pathogen on a birch aphid. Oecologia.

[CR33] Jones RAC (1992). Further studies on losses in productivity caused by infection of annual pasture legumes with three viruses. Aust J Agric Res.

[CR34] Kjaerandsen J, Kurina O, Olafsson E (2007). The fungus gnats of Iceland (Diptera, Keroplatidae, Mycetophilidae). Insect Syst Evol Suppl.

[CR35] Kruess A (2002). Indirect interaction between a fungal plant pathogen and a herbivorous beetle of the weed *Cirsium arvense*. Oecologia.

[CR36] Kurtz B, Karlovsky P, Vidal S (2010). Interaction between western corn rootworm (Coleoptera: Chrysomelidae) larvae and root-infecting *Fusarium verticillioides*. Environ Entomol.

[CR37] Lin L, Shen T-C, Chen Y-H, Hwang S-Y (2008). Responses of *Helicoverpa armigera* to tomato plants previously infected by ToMV or damaged by *H armigera*. J Chem Ecol.

[CR38] Mauck KE, De Moraes CM, Mescher MC (2010). Deceptive chemical signals induced by a plant virus attract insect vectors to inferior hosts. Proc Natl Acad Sci USA.

[CR40] Menzel F, Smith JE, Colauto NB (2003). *Bradysia difformis* Frey and *Bradysia ocellaris* (Comstock): two additional neotropical species of black fungus gnats (Diptera: Sciaridae) of economic importance: a redescription and review. Ann Entomol Soc Am.

[CR41] Menzel F, Smith JE, Chandler PJ (2006). The sciarid fauna of the British Isles (Diptera: Sciaridae), including descriptions of six new species. Zool J Linn Soc Lond.

[CR42] Mewis I, Ulrich Ch, Schnitzler WH (2002). The role of glucosinolates and their hydrolysis products in oviposition and host-plant finding by cabbage webworm, *Hellula undalis*. Entomol Exp Appl.

[CR43] Moran PJ (1998). Plant-mediated interactions between insects and a fungal plant pathogen and the role of plant chemical responses to infection. Oecologia.

[CR44] Mouttet R, Bearez P, Thomas C, Desneux N (2011). Phytophagous arthropods and a pathogen sharing a host plant: evidence for indirect plant-mediated interactions. PLoS One.

[CR45] Najar-Rodriguez AJ, Galizia CG, Stierle J, Dorn S (2010). Behavioral and neurophysiological responses of an insect to changing ratios of constituents in host plant-derived volatile mixtures. J Exp Biol.

[CR46] Norton MR, Johnstone GR (1998). Occurrence of alfalfa mosaic, clover yellow vein, subterranean clover red leaf, and white clover mosaic viruses in white clover throughout Australia. Aust J Agric Res.

[CR47] Pan JJ, Price JS (2002). Fitness and evolution in clonal plants: the impact of clonal growth. Evol Ecol.

[CR48] Pareja M, Moraes MCB, Clark SJ, Birkett MA, Powell W (2007). Response of the aphid parasitoid *Aphidius funebris* to volatiles from undamaged and aphid-infested *Centaurea nigra*. J Chem Ecol.

[CR49] Pierce WH (1935). The identification of certain viruses affecting leguminous plants. J Agric Res.

[CR50] Pieterse CMJ, Dicke M (2007). Plant interactions with microbes and insects: from molecular mechanisms to ecology. Trends Plant Sci.

[CR51] Preston CA, Lewandowski C, Enyedi AJ, Baldwin IT (1999). Tobacco mosaic virus inoculation inhibits wound-induced jasmonic acid-mediated responses within but not between plants. Planta.

[CR52] Quiroz A, Ortega F, Ramirez CC, Wadhams LJ, Pinilla K (2005). Response of the beetle *Hylastinus obscurus* Marsham (Coleoptera: Scolytidae) to red clover (*Trifolium pratense* L) volatiles in a laboratory olfactometer. Environ Entomol.

[CR53] Rasmann S, Köllner TG, Degenhardt J, Hiltpold I, Toepfer S, Kuhlmann U, Gershenzon J, Turlings TCJ (2005). Recruitment of entomopathogenic nematodes by insect-damaged maize roots. Nature.

[CR54] Rayapuram C, Baldwin IT (2008). Host-plant-mediated effects of *Nadefensin* on herbivore and pathogen resistance in *Nicotiana attenuate*. BMC Plant Biol.

[CR55] Renwick JAA (1989). Chemical ecology of oviposition in phytophagous insects. Experientia.

[CR56] Röder G, Rahier M, Naisbit RE (2007). Coping with an antagonist: the impact of a phytopathogenic fungus on the development and behaviour of two species of alpine leaf beetle. Oikos.

[CR57] Röder G, Rahier M, Naisbit RE (2008). Counter-intuitive developmental plasticity induced by host quality. Proc R Soc Lond B.

[CR58] Roossinck MJ (2011). The good viruses: viral mutualistic symbioses. Nature Rev.

[CR59] Rostás M, Simon M, Hilker M (2003). Ecological cross-effects of induced plant responses towards herbivores and phytopathogenic fungi. Basic Appl Ecol.

[CR60] Sackville-Hamilton NRS, Schmid B, Harper JL (1987). Life-history concepts and the population biology of clonal organisms. Philos Trans R Soc Lond B.

[CR61] Saunders JE, Webster JM (2000). Laboratory tests of the susceptibility of some forest insect pests to *Heterorhabditis megidis* H90 (Nematoda). J Invert Path.

[CR62] Schmelz EA, Grebenok RJ, Ohnmeiss TE, Bowers WS (2002). Interactions between *Spinacia oleracea* and *Bradysia impatiens*: a role for phytoecdysteroids. Arch Insect Biochem Physiol.

[CR63] Sherwood RT (1997). Viruses of white clover in pastures of Pennsylvania, New York, and Vermont. Plant Dis.

[CR64] Smart LE, Blight MM (1997). Field discrimination of oilseed rape, *Brassica napus* volatiles by cabbage seed weevil, *Ceutorhynchus assimilis*. J Chem Ecol.

[CR65] Soler R, Harvey JA, Kamp AFD, Vet LEM, Van der Putten WH, Van Dam NM, Stuefer JF, Gols R, Hordijk CA, Bezemer TM (2007). Root herbivores influence the behaviour of an aboveground parasitoid through changes in plant-volatile signals. Oikos.

[CR66] Springer TL, Carlton CE (1993). Oviposition preference of darkwinged fungus gnats (Diptera: Sciaridae) among *Trifolium* species. J Econ Entomol.

[CR67] Stout MJ, Fidantsef AL, Duffey SS, Bostock RM (1999). Signal interactions in pathogen and insect attack: systemic plant-mediated interactions between pathogens and herbivores of the tomato, *Lycopersicon esculentum*. Physiol Mol Plant Pathol.

[CR68] Stout MJ, Thaler JS, Thomma BPHJ (2006). Plant-mediated interactions between pathogenic microorganisms and herbivorous arthropods. Annu Rev Entomol.

[CR69] Takabayashi J, Dicke M (1992). Response of predatory mites with different rearing histories to volatiles of uninfested plants. Entomol Exp Appl.

[CR70] Tapio E (1970). Virus diseases of legumes in Finland and in the Scandinavian countries. Ann Agric Fenn.

[CR71] Thaler JS, Farag MA, Pare PW, Dicke M (2002). Jasmonate-deficient plants have reduced direct and indirect defences against herbivores. Ecol Lett.

[CR72] Thaler JS, Agrawal AA, Halitschke R (2010). Salicylate-mediated interactions between pathogens and herbivores. Ecology.

[CR73] Van Dam NM (2009). How plants cope with biotic interactions. Plant Biol.

[CR74] Van Mölken T, Stuefer JF (2011). The potential of plant viruses to promote genotypic diversity via genotype × environment interactions. Ann Bot.

[CR75] Van Mölken T, Sundelin T, Snetselaar R, Stuefer JF (2011). Highways for internal virus spread: patterns of virus movement in the stoloniferous herb *Trifolium repens*. Botany.

[CR76] Vet LEM, Dicke M (1992). Ecology of infochemical use by natural enemies in a tritrophic context. Annu Rev Entomol.

[CR77] Vilkamaa P, Salmela J, Hippa H (2007). Black fungus-gnats in deciduous forest habitat in northern Europe, with the description of *Bradysia arcula* sp n (Diptera: Sciaridae). Entomol Fenn.

[CR78] Weijschede J, Martinkova J, De Kroon H, Huber H (2006). Shade avoidance in *Trifolium repens*: costs and benefits of plasticity in petiole length and leaf size. New Phytol.

[CR79] Werner BJ, Mowry TM, Bosque-Pérez NA, Ding H, Eigenbrode SD (2009). Changes in green peach aphid responses to *Potato leafroll virus*-induced volatiles emitted during disease progression. Chem Ecol.

[CR80] Winkler E, Fischer M (1999). Two fitness measures for clonal plants and the importance of spatial aspects. Plant Ecol.

[CR81] Wroth JM, Dilworth MJ, Jones RAC (1993). Impaired nodule function in *Medicago polymorpha* L infected with alfalfa mosaic virus. New Phytol.

[CR82] Yin-Quan L, Thiel A, Hoffmeister TS (2009). Odor-mediated patch choice in the parasitoid *Venturia canescens*: temporal decision dynamics. Entomol Exp Appl.

