# Supplementary material for: Virus infection decreases the attractiveness of white clover plants for a non-vectoring herbivore
Source: Oecologia. 2012 Apr 17;170(2):433–44. doi: 10.1007/s00442-012-2322-z (PMC3439618; doi:10.1007/s00442-012-2322-z)
Supplement: Supplementary file 1 — Supplementary material 1 (DOC 62 kb) [file 442_2012_2322_MOESM1_ESM.doc]

# Supplementary Table 1

Values ± SE of various developmental and growth traits in each of the treatments. Pr. Stolon indicates the primary stolon.

|  |  |  |  |  |  |  |  |  |  |  |  |  |  |  |  |  |
| --- | --- | --- | --- | --- | --- | --- | --- | --- | --- | --- | --- | --- | --- | --- | --- | --- |
| Trait |  | Control | | |  | Virus | | |  | Fungus gnats | | |  | Virus + fungus gnats | | |
| Total no. of ramets |  | 55.56 | ± | 2.737 |  | 52.50 | ± | 1.255 |  | 32.38 | ± | 1.775 |  | 27.75 | ± | 2.355 |
| Length of pr. stolon |  | 339.19 | ± | 10.596 |  | 286.94 | ± | 5.646 |  | 289.50 | ± | 12.203 |  | 253.75 | ± | 13.855 |
| % Branches on pr. stolon |  | 59.45 | ± | 2.241 |  | 58.64 | ± | 1.867 |  | 38.73 | ± | 3.252 |  | 31.25 | ± | 3.825 |
|  |  |  |  |  |  |  |  |  |  |  |  |  |  |  |  |  |
| Total biomass |  | 1.36 | ± | 0.073 |  | 0.99 | ± | 0.035 |  | 0.64 | ± | 0.053 |  | 0.48 | ± | 0.051 |
| Biomass of roots |  | 0.26 | ± | 0.012 |  | 0.22 | ± | 0.008 |  | 0.13 | ± | 0.011 |  | 0.10 | ± | 0.013 |
| Biomass of stolons |  | 0.46 | ± | 0.026 |  | 0.31 | ± | 0.012 |  | 0.24 | ± | 0.022 |  | 0.18 | ± | 0.016 |
| Biomass of leaves |  | 0.64 | ± | 0.038 |  | 0.46 | ± | 0.018 |  | 0.27 | ± | 0.022 |  | 0.21 | ± | 0.023 |
| Biomass per ramet |  | 0.02 | ± | 0.001 |  | 0.02 | ± | 0.001 |  | 0.02 | ± | 0.001 |  | 0.02 | ± | 0.001 |
| Root-shoot ratio |  | 0.24 | ± | 0.009 |  | 0.29 | ± | 0.008 |  | 0.27 | ± | 0.012 |  | 0.24 | ± | 0.012 |
|  |  |  |  |  |  |  |  |  |  |  |  |  |  |  |  |  |
| % Biomass of roots |  | 19.59 | ± | 0.597 |  | 22.22 | ± | 0.464 |  | 21.42 | ± | 0.744 |  | 18.82 | ± | 1.033 |
| % Biomass of stolons |  | 33.47 | ± | 0.380 |  | 31.05 | ± | 0.358 |  | 36.80 | ± | 1.013 |  | 37.84 | ± | 1.501 |
| % Biomass of leaves |  | 46.94 | ± | 0.669 |  | 46.73 | ± | 0.543 |  | 41.78 | ± | 0.836 |  | 43.34 | ± | 1.177 |
|  |  |  |  |  |  |  |  |  |  |  |  |  |  |  |  |  |
